# Performance Evaluation of Calypso 4D Localization and Kilovoltage Image Guidance Systems for Interfraction Motion Management of Prostate Patients

**DOI:** 10.1100/tsw.2009.61

**Published:** 2009-06-12

**Authors:** Tomi Ogunleye, Peter J. Rossi, Ashesh B. Jani, Tim Fox, Eric Elder

**Affiliations:** Department of Radiation Oncology, Emory University School of Medicine, Atlanta, GA 30322, USA

**Keywords:** Calypso, prostate, OBI, tracking, motion, electromagnetic localization, real-time

## Abstract

Prostate cancer represents a model site for advances in understanding inter- and intrafraction motion for radiotherapy. In this study, we examined the correlation of the electromagnetic transponder system/Calypso 4D Localization System with conventional on-board imaging (OBI) using kilovoltage imaging. Initially using a quality assurance (QA) phantom and subsequently using data of seven patients, the vector distances between Calypso- and OBI-recorded shifts were compared using the t-test. For the 30 phantom measurements, the average differences between the measured Calypso offset and the calculated OBI shift were 0.4 ± 0.4, 0.2 ± 0.3, and 0.4 ± 0.3 mm in the lateral, longitudinal, and vertical directions, respectively (*p* = 0.73, *p* = 0.91, and *p* = 0.99, respectively), and the average difference vector for all sessions was 0.8 ± 0.4 mm. For the 259 patient measurements, the average differences between the measured Calypso offset and the calculated OBI shift were 0.7 ± 0.5, 1.1 ± 0.9, and 1.2 ± 0.9 mm in the lateral, longitudinal, and vertical directions, respectively (*p* = 0.45, *p* = 0.28, and *p* = 0.56, respectively), and the average difference vector for all sessions was 2.1 ± 1.0 mm. Our results demonstrated good correlation between Calypso and OBI. While other studies have explored the issue of Calypso/OBI correlation, our analysis is unique in our use of phantom validation and in our performing the patient analysis on an initial population prior to routine setup using Calypso without OBI. Implications for Calypso's role as a QA tool are discussed.

### INTRODUCTION

Accurate localization of planning target volumes (PTVs) and critical structures is necessary for today's intensity-modulated radiation therapy (IMRT) plans. These plans incorporate dose distributions with sharp dose falloff and minimal target margin. Alignment of the radiation fields has therefore shifted from positioning the patient to the treatment delivery system's isocenter with the aid of external skin marks and lasers to image-guided techniques visualizing internal structures. Furthermore, for prostate treatments, interfractional migration of PTVs and critical structures has changed the focus for localization from bony landmarks to implanted radio-opaque fiducials.

With the amount of inter- and intrafraction motion that occurs in the prostate, it is necessary to use a form of localization prior to treatment. Studies have shown that use of implanted prostate markers with kilovoltage (kV) or megavoltage (MV) imaging of localization or matching is a preferred, accurate, and reliable method for positioning prostate patients for treatment compared with laser-guided external skin alignment[1,2]. Image-guided radiation therapy (IGRT) utilizing integrated kV imaging with the linear accelerator has been performed in the Department of Radiation Oncology at Emory University since 2004. Implanted gold markers yield high contrast compared to soft tissue and skeletal structures when visualized with the kV imager. Matching of these markers on the kV images to digitally reconstructed radiographs (DRR) created from the planning CT image set can be performed quickly and easily. At the same time, matching of radiographs, whether performed manually or automatically, remains subjective in terms of the quality of the match. Additionally, radiographs provide positional information at an instance in time and cannot be used to monitor intrafractional motion.

The use of an implantable radiofrequency-emitting device has been reported as an alternative to the use of radiographic localization of fiducial markers for prostate patients[3]. With this system, the use of kV or MV imaging may be eliminated for interfraction motion management of the prostate. An additional feature is that this type of electromagnetic localization provides real-time, continuous monitoring of the target position during treatment. This allows the clinician to observe possible intrafraction motion of the prostate for possible tracking of the target.

This report compares radiofrequency-emitting markers with kV imaging localization of markers for evaluation of interfraction motion of the prostate.

### MATERIALS AND METHODS

#### Hardware Localization Systems

The Calypso® 4D Localization System (Calypso Medical Technologies, Seattle, WA) consists of three in-room components: the camera system, the electromagnetic array, and the patient-implanted transponders. An infrared camera system, comprised of three ceiling-mounted cameras aligned with the isocenter geometry, is utilized to localize a 4D electromagnetic array. Infrared beacons on the array allow the camera system to know the position of the array with respect to the LINAC geometry. This array, in turn, communicates with three electromagnetic transponders implanted in the prostate, determining their locations in relation to a planned isocenter to within 0.5 mm[4]. During the treatment planning process, the location of these three radio-opaque transponders with regard to the planned isocenter is determined and entered into the Calypso system. When aligning the patient in the treatment vault, the system uses the measured coordinates of the transponders to position the planned isocenter and the actual isocenter within a fraction of a millimeter[3]. Our system is calibrated monthly using a phantom and consistently shows a system accuracy of 0.3 mm or less.

The use of the Calypso system for positioning the prostate prior to treatment delivery is described in three steps. First, the electromagnetic array is positioned over the patient/phantom on the treatment table so that it can optimally detect the signals from the three transponders while being visualized by the infrared camera system. Once that is achieved, the Calypso system computes the distance and coordinates (lateral, longitudinal, and vertical) between the treatment machine's isocenter and treatment planned position. This allows the user to move the patient or phantom to the planned position. At this stage, the prostate or target volume has been positioned to match the treatment plan and treatment may be initiated. After the localization process is completed, the system can then track the movement of the patient's isocenter in three dimensions in real time with a frequency of 10 Hz.

A Varian 23EX (Varian Medical Systems, Palo Alto, CA) LINAC forms the basis of the integrated on-board imaging (OBI) system. This accelerator is computer controlled and produces 6- and 18-MV photon beams. The OBI system is composed of an X-ray tube (model G242, 0.4- and 0.8-mm focal spots, 14° anode angle, 800 kJ/h, Varian Medical Systems, Inc., Salt Lake City, UT) and an amorphous-silicon imaging panel (model PaxScan® 4030CB, Varian Medical Systems, Inc., Salt Lake City, UT) attached to the gantry of the LINAC with robotic arms. Three motorized pivot points on the robotic arms allow the OBI to be remotely extended or retracted from the control console during patient treatments. The kV X-ray source can be located between 80 and 100 cm from the machine isocenter, and the MV source is fixed at 100 cm from the machine isocenter. With the X-ray collimator fully open and the imager at the maximum distance from the source, the maximum field size at isocenter is $20 \times 27$ cm$^2$.

Orthogonal kV radiographic matching with three implanted radio-opaque markers in the prostate is employed for localization. Anterior-posterior (AP) and right lateral kV images are acquired with the patient in the treatment position and are compared to DRR created from the patient's treatment plan. The user operating the machine acquires kV images of the patient, and performs shifts on the overlaid kV image and DRR to align the markers in both images. The resulting lateral, longitudinal, and vertical shifts are then applied to the treatment table, and treatment delivery is initiated. Studies have shown that a match accuracy of less than 2 mm is attainable with orthogonal kV pair matching with fiducial markers[5]. Orthogonal kV imaging and localization is routinely performed in 3–4 min[6]. Calibration of the OBI localization system is checked weekly and provides an accuracy of 1.0 mm[7].

In this study, both the Calypso and OBI systems are installed on a single linear accelerator, and both systems rely on three markers implanted within the prostate. The Calypso system uses three beacons placed in specific regions of the prostate (apex, left base, and right base), while the OBI system typically employs several gold radio-opaque markers positioned within the prostate[1,4,8,9]. For this study, it was only necessary to implant the Calypso beacons as these markers are radio-opaque and are visualized with kV radiographs. Fig. 1 shows the integrated Calypso and OBI system for our LINAC. The OBI system is used as a reference to compare setup accuracy of the Calypso system. For a stationary target, the measured offset of the target isocenter from the correct position as indicated by the Calypso system should be the exact opposite of the OBI shift required to move the target isocenter to the correct position. Stated differently, if the Calypso system specifies a 5-mm lateral offset in the +X direction from the correct position, the OBI system should indicate a 5-mm lateral offset in the –X direction to align the target with the real isocenter correctly. The Calypso system measures misalignment, while the OBI system provides the required values to realign.

### Phantom Measurements

A quality assurance (QA) phantom, as seen in Fig. 2, is used for confirming the calibration of the system each day of treatment – comparing the Calypso-calculated machine center of treatment to the one defined by the room lasers and machine collimator cross-hairs. A CT imaging study of the phantom containing three electromagnetic beacons was acquired with 1.25-mm axial slices. A treatment plan was created using the centroid of the three beacons as the planning isocenter or target point. For this study, 30 phantom positions were chosen by randomly shifting the lateral, longitudinal, and vertical directions. Each new position consisted of shifts in all three directions. The magnitude of the planned isocenter to actual isocenter offset in any one direction was less than 20 mm. The Calypso offset coordinates were recorded after each positioning of the phantom. AP and lateral kV images were then acquired and a visual match was performed, aligning the beacons on the DRRs to those on the kV radiographs.

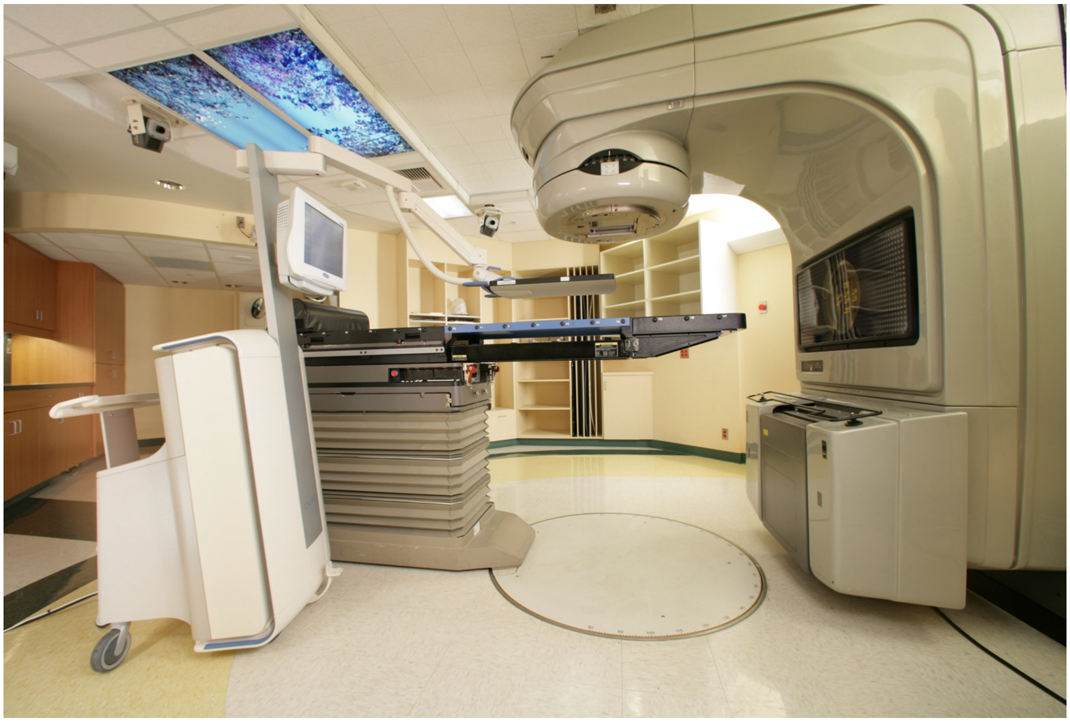

**FIGURE 1.** Calypso Localization System installed on a Varian LINAC. (Photo courtesy of Calypso Medical Technologies, Inc.)

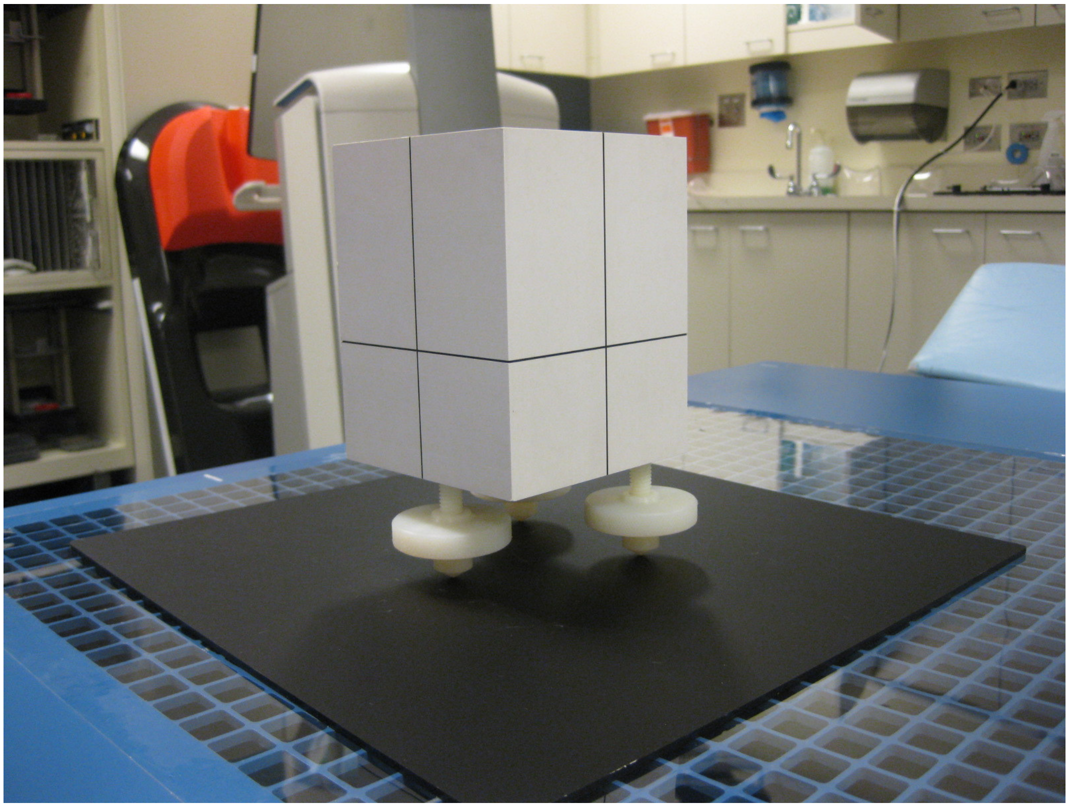

**FIGURE 2.** Calypso QA phantom with implanted transponder beacons used for daily calibration of isocenter.

### Patient Measurements

Seven patients were implanted with transponders for this study. Shifts were monitored and performed during 259 patient sessions. During each patient session, the patient was positioned using the treatment room lasers and the linear accelerator light field. The Calypso system was moved into position and real-time monitoring of the planned isocenter to actual isocenter offset was initiated. After exiting the treatment delivery room, the therapists prepared the OBI system for acquisition of the orthogonal radiographs. Immediately prior to taking the radiographs, the Calypso offsets were recorded. After completing the orthogonal images, a radiograph-DRR matching was performed using the radio-opaque Calypso beacons as the alignment points.

The OBI calculated shifts were recorded for the radiographic localization coordinates.

### Data Evaluation

The distance between the Calypso and OBI recorded shifts was used to evaluate the accuracy of the Calypso system. The difference vector for each position is defined by:

$$Difference\quad Vector = \sqrt{(Cx - Ox)^2 + (Cy - Oy)^2 + (Cz - Oz)^2} \qquad (1)$$

where the subscripts x, y, and z represent the Calypso (C) and OBI (O) lateral, longitudinal, and vertical values, respectively.

## RESULTS

### Phantom Measurements

The radiographs and DRRs of the phantom provided clear visibility of the Calypso beacons, allowing alignment of the images. The bottom left quadrant of Fig. 3 shows the AP DRR and the AP OBI image on the left and right, respectively. Note the Calypso electromagnetic array on the radiograph. The bottom right quadrant demonstrates the lateral images. The upper portion of Fig. 3 shows the blended AP and RT lateral views after the radiographs and DRRs have been correctly aligned.

For the 30 phantom positions, the average differences between the measured Calypso offset and the calculated OBI shift were 0.4 ± 0.4, 0.2 ± 0.3, and 0.4 ± 0.3 mm in the lateral, longitudinal, and vertical directions with $p$-values $p = 0.73$, $p = 0.91$, and $p = 0.99$, respectively. The magnitudes of the individual values are shown in Figs. 4A–C. The average difference vector for all 30 sessions was 0.8 ± 0.4 mm. As stated earlier, the Calypso system has a localization resolution of 0.5 mm. Although we did not set up our sample size of 30 data points to reach a specific statistical end point, the sample size produced a standard deviation of less than 0.5 mm for each of the directional components of the system. A standard deviation smaller than the system resolution gives us confidence that our data yielded a good representation of the range of values of difference between the Calypso and OBI system for a phantom setup.

### Patient Measurements

The Calypso system offsets and corresponding OBI shift were compared for 259 patient sessions. Figs. 5A–C show the Calypso system and OBI offset and shift data for each axis of measurement for one patient. Calypso-OBI comparison was not performed during fractions 8 and 9. For this patient, the average difference between the Calypso system offsets and the OBI shifts for the lateral, longitudinal, and

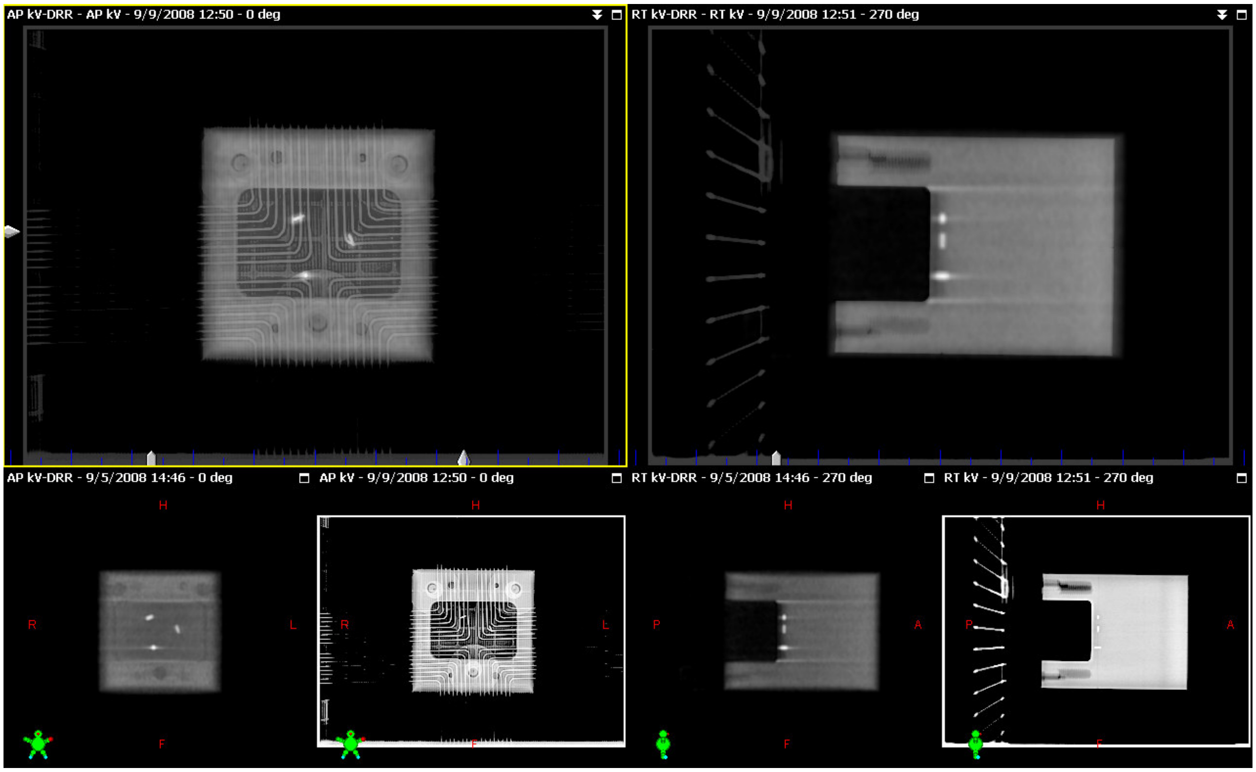

**FIGURE 3.** kv-kv Matching user interface showing blended images in AP and lateral views for Calypso phantom.

vertical directions were 0.8, 0.7, and 1.1 mm, respectively. The average difference vector magnitude is 1.8 mm. The histogram in Fig. 6 represents 259 session measurements. The components of Calypso system offsets and OBI shifts differed by an average of 0.7 ± 0.5, 1.1 ± 0.9, and 1.2 ± 0.9 mm in the lateral, longitudinal, and vertical directions, respectively ($p$ = 0.45, $p$ = 0.28, and $p$ = 0.56, respectively), and the average difference vector for all sessions was 2.1 ± 1.0 mm.

### DISCUSSION

The results from our study show that the Calypso Localization System and kV image guidance matching correlate well with each other. For the measurements in this study, the maximum vector difference magnitude between the two systems was less than 5.5 mm, with an average difference of 2.1 ± 1.0 mm. Other studies have compared the Calypso tracking system with kV localization systems[10,11]. Jani et al. performed similar tests for prostate patients using the ExactTrac X-ray system from BrainLab. Their results show that the average vector length between the two systems was 1.9 ± 1.2 mm[11].

Other studies have noted the potential uncertainties in correlating Calypso vs. X-ray systems because of the possibility of target motion during imaging intervals[12]. In an attempt to reduce this uncertainty, the initial offsets from the session were not recorded until just prior to acquisition of the first orthogonal pair of kV images. As expected, the shift expected by Calypso and the actual OBI shift generated from analyzing the radiographs correlated well.

Although prior studies explored the issue of correlation of OBI with Calypso, our study is unique. First, we systematically reported the results using a phantom initially prior to patient data, and we demonstrated similar results for patient and phantom data. Second, our study is a correlative analysis of OBI with Calypso for an initial patient population prior to implementing Calypso without OBI on routine follow-up. Although

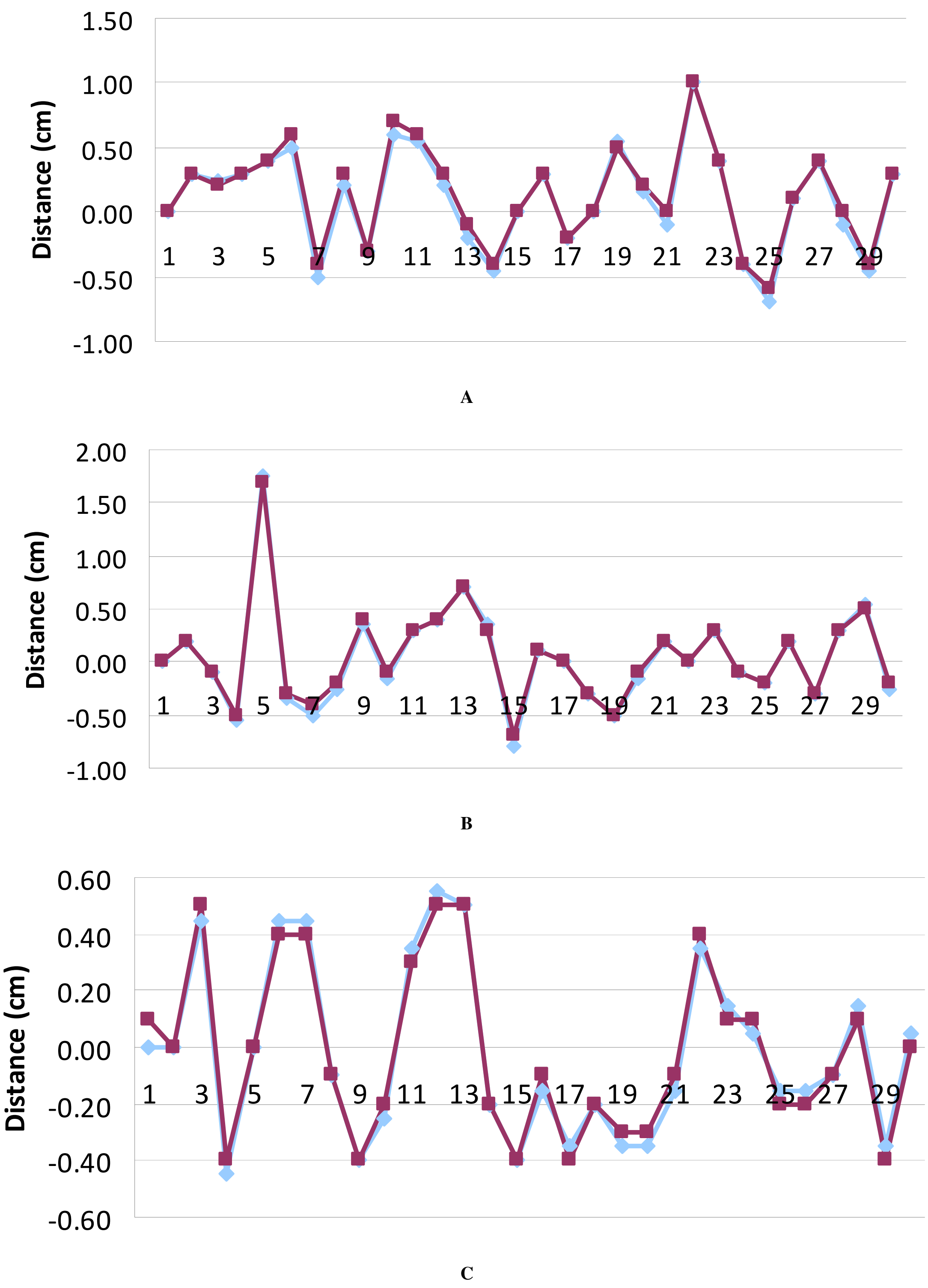

**FIGURE 4.** Comparison of (A) lateral, (B) longitudinal, and (C) vertical values of Calypso offset and OBI shift through 30 sessions with Calypso phantom. –♦– Calypso offset; –■– OBI shift.

we found excellent correlation between Calypso and OBI, our method could potentially identify systematic errors and would be a means of commissioning Calypso prior to its routine use without OBI.

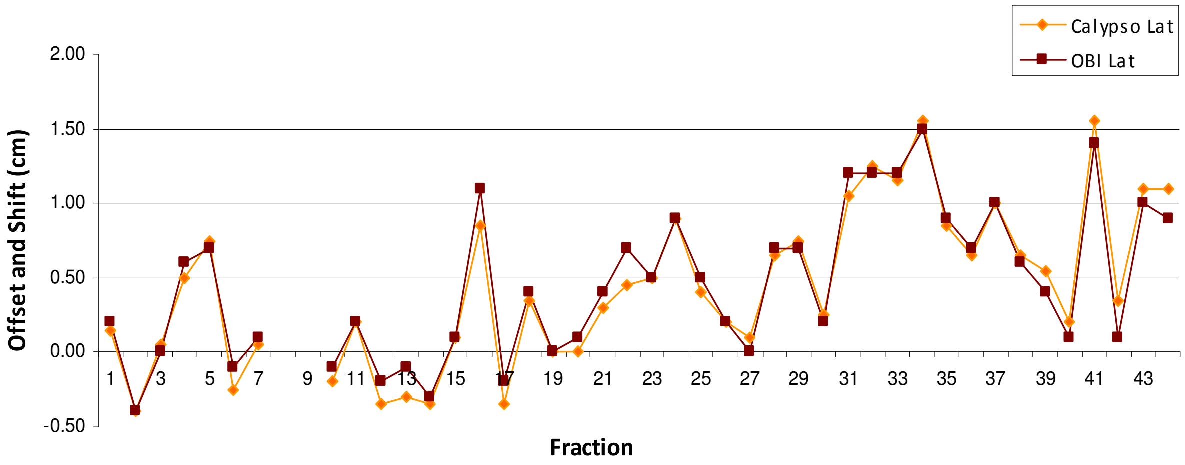

A

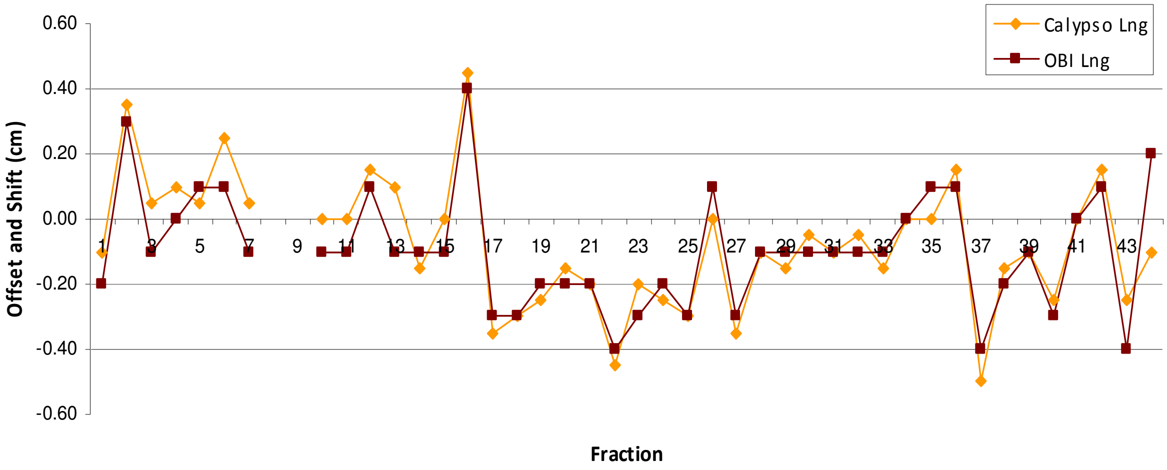

B

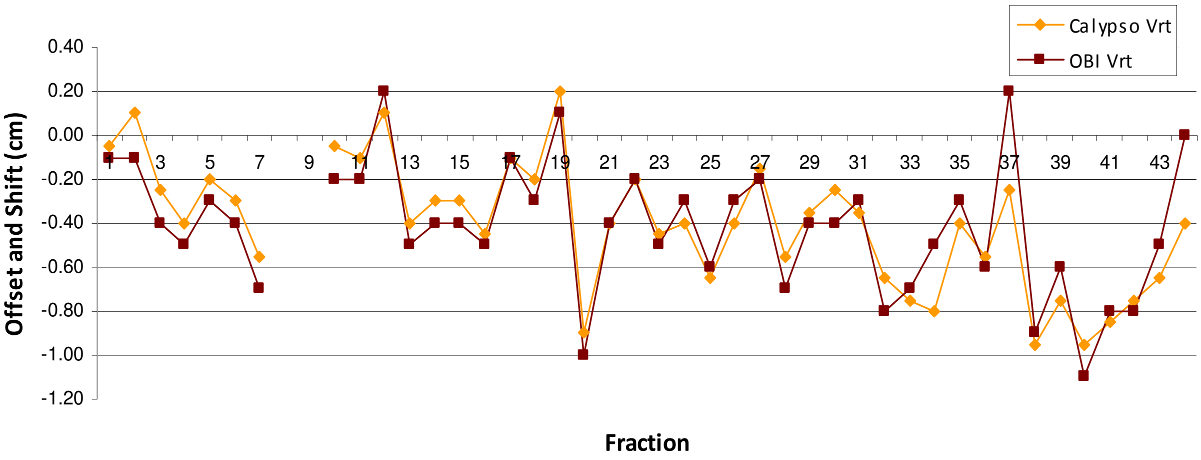

C

**FIGURE 5.** Comparison of (A) lateral, (B) longitudinal, and (C) vertical values of Calypso offset and OBI shift at each treatment session for a patient.

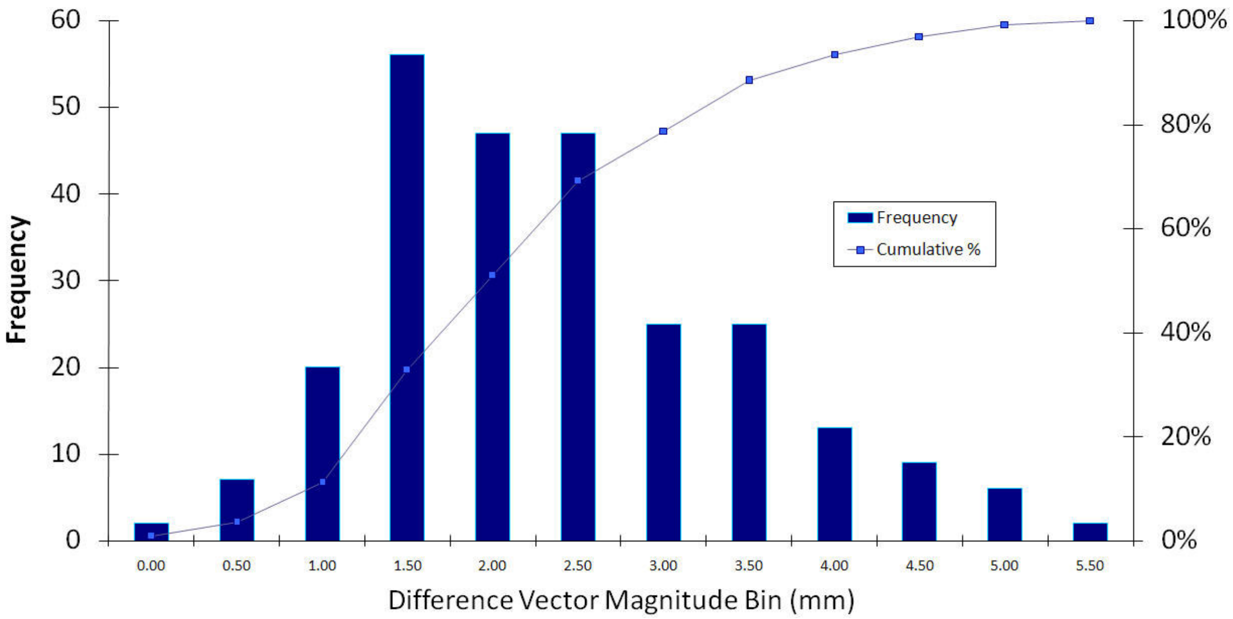

**FIGURE 6.** Calypso-OBI difference vector magnitude histogram through 259 localization sessions.

We also observed motion of the prostate during treatment by using the Calypso system's ability to track real-time movement of the beacons. Information on the maximum and average deviations from baseline was determined for each session using the absolute value of the tracking data in the X (lateral), Y (longitudinal), and Z (vertical) directions. Daily averages and maximum values were used to determine the averages for the entire treatment course for each patient, as well as for all treatment sessions.

There were three categories of intrafraction motion observed during this study. In one case, the prostate remained stable, within 2.0 mm of the localized position for the duration of treatment. In the second category, the prostate started out in the correct setup position, but gradually drifted away from the localized position over the course of the treatment fraction. In the third category, the prostate had several short excursions from the area within the tracking limits. In a few of the tracking sessions in this category, the patient coughed during treatment, which resulted in large movements in the longitudinal and vertical directions.

In a sampling of 268 tracking sessions, the average intrafraction prostate motion for all sessions was 0.35 mm (range: 0.06–2.0) in X, 0.87 mm (range: 0.15–3.7) in Y, and 0.96 mm (range: 1.3–6.7) in Z directions. Average maximum motion for all sessions was 1.3 mm (range: 0.2–5.5) in X, 3.5 mm (range: 0.6–14.1) in Y, and 3.8 mm (range: 0.4–50.5) in Z directions, respectively[13].

### CONCLUSION

This analysis of Calypso system offsets and kV image guidance shifts showed good correlation between the two systems. The information from our tracking sessions as well as other studies show that prostate motion is least in the lateral direction[12,14]. Therefore, it is not surprising that the average difference between these two systems is the lowest (0.75 mm) in the lateral direction. Our evaluation shows that the Calypso system provides a similar level of confidence in localization for prostate patients without the need for daily orthogonal kV imaging, while also providing an objective measurement of patient position and target movement during treatment.
